# Hemophagocytic Lymphohistiocytosis Triggered by Dengue: A Narrative Review and Individual Patient Data Meta-Analysis

**DOI:** 10.3390/v17081047

**Published:** 2025-07-27

**Authors:** Angelos Sourris, Alexandra Vorria, Despoina Kypraiou, Andreas G. Tsantes, Petros Ioannou

**Affiliations:** 1 School of Medicine, University of Crete, 71003 Heraklion, Greece; 2 Laboratory of Hematology and Blood Bank Unit, “Attikon” University Hospital, School of Medicine, National and Kapodistrian University of Athens, 12462 Athens, Greece

**Keywords:** dengue virus, hemophagocytic lymphohistiocytosis, HLH, secondary HLH

## Abstract

Background: Hemophagocytic lymphohistiocytosis (HLH) is a life-threatening hyperinflammatory syndrome that may be triggered by infections such as dengue virus. Due to overlapping features with severe dengue and sepsis, diagnosis of HLH in dengue-infected patients remains challenging. Methods: We conducted a narrative review and individual patient data meta-analysis of published cases of dengue-associated HLH. Eligible studies were identified through a search of PubMed and Scopus databases up to 5 March 2025. Clinical, laboratory, microbiological, treatment, and outcome data were extracted and analyzed. Results: A total of 133 patients from 71 studies were included. The median patient age was 18 years, and 56.8% were male. Common clinical features included fever (96.9%), cytopenias, organomegaly, and liver dysfunction. ALT elevation, jaundice, and hypofibrinogenemia were associated with mortality. DENV-1 was the most common serotype (57.4%) and was negatively associated with death. Overall, 19.3% of patients died. Multivariate analysis did not identify independent mortality predictors. Conclusions: Dengue-associated HLH predominantly affects young individuals and carries significant mortality. Key indicators of poor prognosis include hepatic dysfunction and the presence of shock or organ failure. Early recognition and prompt immunomodulatory treatment, particularly corticosteroids, may improve outcomes.

## 1. Introduction

Hemophagocytic lymphohistiocytosis (HLH) is a clinical syndrome that can be proven fatal due to its non-specific clinical presentation and sepsis-like signs and symptoms where patients are unresponsive to common therapies affecting both children and adults. A constellation of nonspecific signs of systemic inflammation, such as prolonged fever, cytopenias, hepatosplenomegaly, and elevated inflammatory markers like ferritin, in combination with tissue damage expressed by neurological symptoms, liver dysfunction, myelosuppression due to hemophagocytosis, and respiratory distress, often rise clinicians’ suspicion about HLH. This clinical entity can be characterized either as primary, where genetic errors result in a broader dysregulation of cytotoxic T cells, NK cells and macrophages, combined with hypercytokinemia, resulting in overstimulation of the immune system, or as secondary [1,2,3]. Secondary HLH is the manifestation of the host’s exposure to a trigger, that can be either infectious, malignancy related, or associated with an autoimmune disease. As far as infectious triggers are concerned, viral infections (especially EBV) and bacterial and fungal infections are enlisted [4,5]. Among viral infections, dengue virus seems to play a significant role as a trigger of secondary HLH. More specifically, the prevalence of dengue-associated HLH can be up to 22.1% in patients with severe dengue, while the mortality rate is high (20.2%) [6].

Dengue virus is an RNA virus that belongs to the broader family of Flaviviridae. It is primarily transmitted by *Aedes aegypti* and *Aedes albopictus* mosquitoes and is endemic mainly in tropical and subtropical regions. There are four distinct serotypes (DENV-1 to DENV-4) based on antigen expression, and the clinical manifestation of the infection can range from either asymptomatic (40–80% of d infections) or symptomatic. Symptomatic dengue or dengue fever is a mild to moderate acute febrile illness with non-specific signs and symptoms. A small proportion of patients (<5%) develop severe and life-threatening disease in the form of dengue hemorrhagic fever and dengue shock syndrome which is characterized by increased vascular permeability, thrombocytopenia, and potentially life-threatening shock [7,8].

The aim of the present review is to summarize the existing data regarding HLH secondary to dengue fever in a comprehensive way, by presenting the clinical and laboratory characteristics of this condition with an analysis based on individual patient data.

## 2. Materials and Methods

This narrative review extracted and collected data regarding HLH syndrome associated with dengue cases in humans. The primary objective of the present study was to provide information regarding the epidemiology and the mortality of this infection. Presenting data on (a) the type of dengue, (b) the patients’ clinical characteristics, (c) the diagnostic criteria of HLH, and (d) their treatment were among the secondary outcomes of this study. For this review, Scopus and PubMed/Medline databases were searched for eligible articles reporting “Hemophagocyt* AND dengue” until 5 March 2025. Inclusion criteria for this review included (a) primary research papers such as observational studies, case reports, case series, and RCTs that provided data at least about epidemiology, clinical and microbiological criteria, and outcomes on HLH associated with dengue infection in humans. Papers not in the English language were excluded. Exclusion criteria included studies on animals, secondary papers such as reviews and meta-analyses, and studies not referring to cases of HLH associated with dengue in humans. Additionally, perspectives, editorials, and papers not reporting results of primary research results were excluded. Finally, studies providing aggregated data that were inappropriate for individual patient data meta-analysis were excluded. The remaining articles were examined following the snowball procedure to assess potential studies.

The collected data included publication year; country of origin; study design; demographic details of patients such as age and gender; relevant medical history (drug-induced or primary immunosuppression, autoimmune disease, malignancy, previous history of HLH); clinical symptoms until diagnosis such as fever, jaundice, lymphadenopathy, splenomegaly or/and hepatomegaly; laboratory values and microbiology values (type of dengue, method of identification, concomitant infection); treatment administered; and outcomes (i.e., complications, cure, or death). The association of mortality with the HLH due to dengue infection and causal microbiology was reported by the study authors. In each case, the diagnosis of HLH was confirmed by the investigators of the current study, based on the data provided by the authors in each study and the diagnostic guidelines for HLH presented by the Histiocyte Society in 2004, which require that five out of the eight criteria be fulfilled, given that there are no specific criteria for the diagnosis of infection-associated HLH [9,10].

In the present review, data are presented in numbers (%) for categorical variables and in median (IQR) for continuous variables. Continuous variables were compared using the Student *t*-test for normally distributed variables and the Mann–Whitney U test for non-normally distributed variables. Categorical variables were compared using Fisher’s exact test due to the relatively small sample. A univariate logistic regression analysis was conducted to explore several factors associated with mortality, including gender, age, the presence of immunosuppression status, dengue type, laboratory results, number of diagnostic criteria, and treatment. Subsequently, a multivariable logistic regression analysis was performed on variables that showed statistical significance. All the above-mentioned statistics were calculated with SPSS version 25.0 (IBM Corp., Armonk, NY, USA). All tests were two-tailed, and *p*-values < 0.05 were considered statistically significant.

## 3. Results

### 3.1. Included Studies’ Characteristics

**Figure 1:**
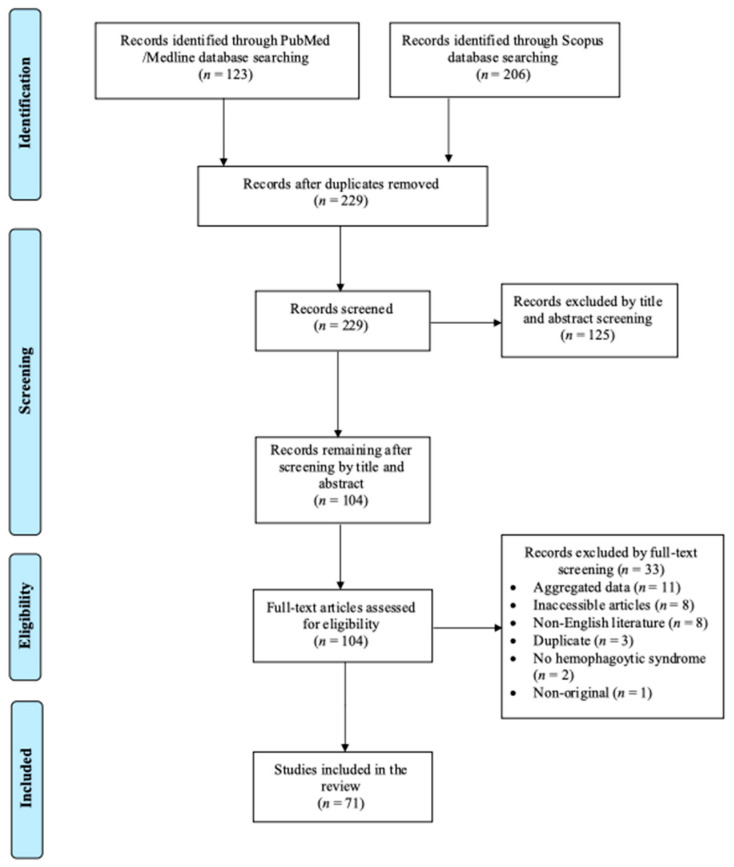
Flow diagram of study inclusion.

A total of 229 articles from Pubmed and Scopus were screened after exclusion of duplicates. Finally, 71 were included in the present analysis [11,12,13,14,15,16,17,18,19,20,21,22,23,24,25,26,27,28,29,30,31,32,33,34,35,36,37,38,39,40,41,42,43,44,45,46,47,48,49,50,51,52,53,54,55,56,57,58,59,60,61,62,63,64,65,66,67,68,69,70,71,72,73,74,75,76,77,78,79,80,81]. These studies involved 133 patients in total. Among these studies, 62 were conducted in Asia, 5 in North and South America, and 4 in Europe. Figure 1 shows the flow diagram of study inclusion.

### 3.2. Epidemiology and Clinical Characteristics of HLH Due to Dengue

The median age of patients with HLH due to dengue is 18 years (range: neonates to 72 years). Most patients were male (75 out of 132 patients with available data). Among the 105 patients with available data, only three had an immunosuppressing medical condition and only one had a history of a previous HLH. Drug-induced immunosuppression was noted in five patients, with corticosteroids, cyclosporine, and mycophenolate mofetil being prescribed more commonly.

The most common presenting symptoms and signs were fever, organomegaly, gastrointestinal symptoms (such as diarrhea, nausea, and vomiting), abdominal pain, respiratory symptoms (such as tachypnea and dyspnea), and skin rash. All patients had thrombocytopenia, most had anemia, and more than half had leukopenia. Most patients had abnormal liver chemistry tests, with aspartate Aminotransferase (AST) and alanine aminotransferase (ALT) being increased in most of the patients. Ferritin was increased in almost all patients, as only four out of 111 patients had a normal ferritin value. Table 1 shows the characteristics of patients with HLH due to dengue, as well as a comparison between patients who survived with those who died.

### 3.3. Microbiology and Diagnosis of HLH Due to Dengue

Among the 115 patients with available data, the diagnosis of dengue was performed with samples from the peripheral blood in 107 (93%); the bone marrow in 4 (3.5%); the blood and the bone marrow in 2 (1.7%); the tissue and the blood in 1 (0.9%); and the blood, the cerebrospinal fluid, and the vesicular fluid in 1 (0.9%). For the diagnosis, serology, most commonly with ELISA, was performed in 96 out of 111 patients with available data (86.5%), while polymerase chain reaction (PCR) was performed in 33 (29.7%). In 47 patients, the type of dengue was mentioned, and was dengue virus-1 (DENV-1) in 27 (57.4%), DENV-2 in 3 (6.4%), DENV-3 in 8 (17%), and DENV-4 in 9 (19.1%). A polymicrobial infection was diagnosed in 15 out of 112 patients with available data (13.4%). The other pathogens were *Aspergillus* spp. in 4 out of 15 patients (26.7%), *Plasmodium falciparum* in 2 (13.3%), severe acute respiratory syndrome virus-2 (SARS-CoV-2) in 2 (13.3%), and 1 (6.7%) in each one of the following: *Brucella* and SARS-CoV-2, *Candida tropicalis* and hepatitis E, *Plasmodium vivax*, scrub typhus, *Staphylococcus hominis* and *Acinetobacter baumannii*, typhus, and, finally, West-Nile virus.

**Figure 2:**
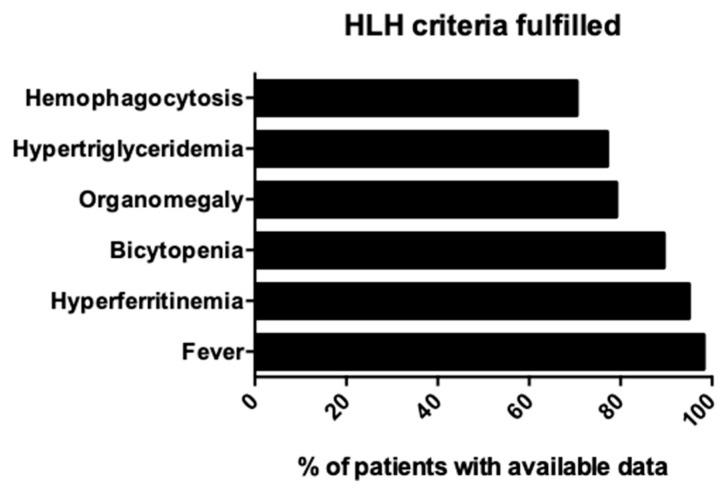
Frequency of criteria fulfilled for the diagnosis of HLH. HLH: hemophagocytic lymphohistiocytosis; data are among patients with available data. Data regarding low or absent NK-cell activity and high levels of sIL-2r are not shown due to inconsistent reporting and unavailable data in the vast majority of studies.

Regarding the diagnostic criteria that aided in the diagnosis of HLH, the most common criterion was fever, which was present in 98.1%; increased ferritin in 94.9%; bicytopenia in 89.4%; organomegaly in 79%; hypertriglyceridemia in 77%; hemophagocytosis in bone marrow, spleen, or lymph nodes in 70.4%; low or absent NK-cell activity in 2.3%; and high levels of soluble interleukin-2 receptor in 2.3%. Figure 2 shows the frequency of criteria fulfilled for the diagnosis of HLH.

### 3.4. Treatment and Outcomes of HLH Due to Dengue

The complications, treatment, and outcome of patients with HLH due to dengue is described in Table 1. Empirical antibiotic treatment was provided in 21.9%. Intravenous immunoglobulin was given to 21.8%, cyclosporine to 2.8%, etoposide to 6.6%, steroids to 64.2%, more commonly as dexamethasone (37%), of methylprednisolone (23%). Fresh frozen plasma was given to 18.3% of patients, plasma exchange to 4.8%, methotrexate to 1%, and anakinra to 1%.

Fulfillment of the Systemic Inflammatory Response Syndrome (SIRS) criteria were noted in 53.8% of patients with dengue and HLH, while shock developed in 30.6%, and 51.4% of patients developed organ dysfunction of at least one organ. Mortality was 19.3% (21 out of 109 patients with available data).

### 3.5. Statistical Analysis of HLH Due to Dengue

Table 1 shows a statistical comparison of patients with HLH due to dengue who survived and those who died. Patients who died were more likely to present with jaundice, and lethargy or drowsiness. Additionally, they had a less pronounced drop in white blood cells, but had more increased ALT, and lower fibrinogen. Patients who died were less likely to have dengue-1 infection, and were more likely to have sepsis, shock, and organ dysfunction, and to need intensive-care-unit level of care.

A univariate linear regression analysis of mortality with all the parameters shown in Table 1 was conducted and identified a positive association between mortality and immunosuppression (*p* = 0.0485), drug-induced immunosuppression (*p* = 0.027), jaundice (*p* < 0.0001), lethargy or drowsiness (*p* = 0.023), increased AST (*p* = 0.0009), increased ALT (*p* < 0.0001), polymicrobial infection (*p* = 0.0368), sepsis (*p* = 0.0009), shock (*p* < 0.0001), organ dysfunction (*p* = 0.0022), need for ICU (*p* < 0.0001), empirical antibiotic therapy (*p* = 0.0421), and plasma exchange (*p* = 0.0242). Additionally, the regression analysis identified a negative association between mortality and dengue-1 diagnosis (*p* = 0.0175). A multivariate logistic regression model did not identify any factors independently associated with mortality.

## 4. Discussion

This narrative review presents individual patient data of patients suffering from dengue virus infection and secondary HLH, and provides insights into the epidemiology, clinical features, diagnostics, treatment, and outcomes associated with the severe complication of HLH in the context of this infection. Through the synthesis of 133 individual cases, this study delivers important evidence to aid clinical recognition and management of dengue-associated HLH, particularly in endemic regions where early diagnosis and treatment are crucial.

A major finding of this review is the younger age distribution among patients who developed HLH following dengue infection. The median age was 18 years, with cases ranging from neonates to 72 years. This is in contrast to literature regarding HLH in general, where the syndrome more commonly affects older adults, particularly those over 65 years of age [82]. The younger age distribution in the present review is likely reflective of the underlying epidemiology of dengue, which is more prevalent among younger individuals in endemic areas. Moreover, the age-related vulnerability to severe dengue seen at both ends of the age spectrum could account for the inclusion of both neonates and older patients among reported cases [83].

Interestingly, the present review also identified a predominance of male patients (56.8%), although male gender was not statistically associated with increased mortality. In fact, mortality was somewhat lower in males than in females, a finding that diverges from broader HLH epidemiology where males often have worse outcomes [82]. This suggests that in dengue-associated HLH, the immunopathogenic mechanisms may override demographic risk factors typically associated with HLH, or that health-seeking behavior and access to care differ by gender in the affected regions.

This review did not find a significant association between immunosuppression and the development of HLH secondary to dengue. Only a small subset of patients had known immunosuppressive conditions or treatment, and these individuals were not disproportionately represented among the deceased. This observation aligns with prior reviews which suggest that, unlike other viral triggers of HLH (e.g., EBV or CMV), dengue may not require a background of immunosuppression to precipitate the hyperinflammatory cascade characteristic of HLH [35]. Nevertheless, other literature has noted associations between immunosuppression and both dengue severity and HLH, suggesting that different pathogenic mechanisms may be at play when immunosuppression is present [84,85]. To that end, the role of previous treatment with corticosteroids or other immunosuppressive medications could be hypothesized to be contributing to a higher likelihood of sepsis symptoms and mortality; however, this study did not identify a higher rate of previous immunosuppression in patients with HLH due to dengue who died compared to those who lived.

Dengue serotyping was reported in about one-third of cases, with DENV-1 being the most prevalent, followed by DENV-3, DENV-4, and DENV-2. Interestingly, DENV-1 infection was less commonly noted among patients who died, even though this should be interpreted with caution, due to the small patient sample. This may indicate it may be a less virulent serotype in the context of HLH, or that that it could possibly be associated with more timely clinical recognition and treatment. This warrants further investigation, especially since previous literature has offered inconsistent data on serotype-specific outcomes [86]. It is worth mentioning that serotype distribution can vary geographically, and the data in the present review were heavily skewed toward Asia, with minimal representation from Africa and North and South America, despite the fact that dengue virus is also endemic there [87]. This may highlight a major gap in global surveillance and can possibly suggest underrecognition or underreporting of HLH in those regions. Notably, since the data presented in the current study are derived from countries where dengue is endemic, little can be said regarding the possibility of diagnosis of dengue-associated HLH in patients residing in non-endemic countries, such as in Europe. Thus, the characteristics and the serotypes associated with dengue-associated HLH in European countries where reports of dengue are rare, and associated with returning travelers, are anticipated to depend on the country where the dengue was acquired. However, the potential of transmission of autochthonous *Aedes*-borne arboviruses, such as dengue, in Europe, has been described, underlying the need to keep a level of awareness for this infection and its complications [88,89].

The hallmark symptoms of HLH in the present review were fever, thrombocytopenia, anemia, and leukopenia. Hepatosplenomegaly, skin rash, respiratory symptoms, and gastrointestinal complaints were also commonly reported. Notably, jaundice and lethargy/drowsiness were significantly more common in patients who died, implying that these features could be signs of a poor prognosis. From a laboratory perspective, elevated liver enzymes (AST and ALT), hyperferritinemia, and hypofibrinogenemia were notable, especially in patients who died. Among these, ALT was significantly elevated in deceased patients, underscoring hepatic dysfunction as a critical factor in disease severity and outcome [90]. Lower fibrinogen levels and increased ferritin also correlated with mortality, consistent with HLH pathophysiology involving cytokine-induced liver injury and coagulopathy [91]. Elevated white blood cell counts in deceased patients compared to survivors may reflect a secondary bacterial infection or a more fulminant inflammatory response.

The diagnosis of HLH was confirmed using the HLH-2004 criteria, which require five of eight features including fever, cytopenias, hyperferritinemia, organomegaly, hypertriglyceridemia, hemophagocytosis, low/absent NK-cell activity, and elevated soluble IL-2 receptor levels. In this review, the most frequently fulfilled criteria were fever, increased ferritin, and cytopenias. Molecular markers such as NK-cell activity and soluble IL-2 receptor were rarely used (<3%), reflecting the high cost and technical limitations in low-resource settings where dengue is prevalent. Even though newer criteria have been developed ever since (HLH-2024 criteria), using these criteria was deemed more appropriate, given that most cases had been published while the previous ones were in use [92].

Peripheral blood was the most common sample used for DENV diagnosis (93%), with ELISA employed in the majority of cases. While ELISA offers logistical and financial advantages, particularly in endemic and resource-limited settings, it also presents limitations including cross-reactivity with other flaviviruses or endemic infections such as malaria and leptospirosis. PCR, used in 29.7% of cases, offers higher specificity but is costlier and less widely available. Currently, novel diagnostic tools with variable availability in different countries are available, or under evaluation, targeting dengue antigens, host antibodies, or the genetic material of the virus, providing better diagnostic ability [93].

Treatment regimens varied widely in the present review, reflecting the lack of consensus guidelines for managing dengue-associated HLH. Corticosteroids were the cornerstone of therapy, administered to 64.2% of patients, with dexamethasone being the most common agent. Steroids are a mainstay of HLH treatment due to their immunosuppressive effects, and their frequent use here underscores their perceived efficacy. Intravenous immunoglobulin (IVIG) was given to 21.8% of patients, and while its role in secondary HLH is debated, it remains a part of many institutional protocols [94,95]. Empirical antibiotics were administered in 28.7% of cases, a decision likely driven by clinical presentations indistinguishable from sepsis and the high rate of polymicrobial infections (13.4%). This is also seen in patients treated with dengue shock, where high rates of antibiotic use are noted, often being inappropriate [96]. However, the association between empirical antimicrobial use and mortality may result from more severe co-infections. Similarly, the high frequency of corticosteroid use may reflect greater disease severity. Plasma exchange and cytotoxic agents like etoposide were less frequently used, reflecting their toxicity profiles and the difficulty of administering such treatments in resource-constrained environments.

Complications were common and often severe. Over half of the patients developed sepsis or organ dysfunction, and nearly one-third developed shock. ICU admission was required in more than one out of five patients, and the overall case fatality rate was 19.3%, consistent with prior systematic reviews [97,98]. These figures underscore the seriousness of HLH as a dengue complication and the urgent need for early recognition and intervention. Univariate regression analysis identified multiple factors positively associated with mortality, including immunosuppression, jaundice, lethargy, elevated liver enzymes, polymicrobial infection, and shock. Conversely, infection with DENV-1 was associated with reduced mortality. However, the multivariate analysis that was performed with the individual patient data failed to confirm any independent predictors of mortality, likely due to the limited sample size and heterogeneity in data collection.

There is a clear need for standardized diagnostic and treatment protocols tailored to resource-limited settings. Further prospective studies are necessary to identify the role of different dengue serotypes in the pathogenesis of HLH and to identify reliable prognostic markers. Additionally, investment in molecular diagnostic infrastructure and training could facilitate earlier and more accurate diagnoses. Finally, this study highlights the need for enhanced global surveillance, particularly in underrepresented regions such as Africa and the Americas, to better characterize the true burden of dengue-associated HLH and inform public health responses.

Several limitations must be acknowledged. First, the inherent nature of narrative reviews and individual patient data meta-analyses introduces potential biases, particularly due to the reliance on published case reports and series, which may selectively report more severe or unusual cases of dengue-associated HLH. This selection bias limits the generalizability of the findings to broader populations. Additionally, due to the need for individual patient data, studies providing aggregated data were excluded. Second, a significant proportion of included studies lacked complete datasets, with missing values for key clinical and laboratory parameters. For instance, dengue serotyping was reported in less than one-third of patients, and molecular HLH diagnostic markers such as NK cell activity and soluble IL-2 receptor levels were infrequently measured. This limitation impairs the ability to perform more granular subgroup analyses and may underestimate the contribution of certain risk factors. Third, geographic representation of patients was skewed towards Asia, with minimal data from Africa and North and South America. Given the fact that dengue virus is endemic in these regions, the underrepresentation may reflect systemic underreporting or diagnostic challenges rather than true epidemiological trends. Consequently, the findings may not fully capture regional variability in clinical presentation, serotype distribution, or outcomes. Fourth, due to the retrospective and observational nature of the source studies, heterogeneity in diagnostic criteria, treatment approaches, and reporting standards across different centers and countries could not be controlled. This variability may affect the consistency and comparability of the data presented herein, particularly with regard to treatment outcomes and mortality predictors. Lastly, even though univariate regression analysis identified several variables associated with mortality, the multivariate analysis did not confirm any independent predictors. This may be due to the limited sample size and statistical power, suggesting that some potentially meaningful associations could not be detected with the present data. Future prospective, multicenter studies with standardized data collection, broader geographic inclusion, and consistent diagnostic protocols are warranted to validate and extend the findings of this review.

## 5. Conclusions

Dengue-associated HLH is a rare but life-threatening condition that poses significant diagnostic and therapeutic challenges. Recognition of key clinical and laboratory features, prompt diagnosis using available criteria, and early initiation of appropriate therapy, particularly corticosteroids, are essential to improving outcomes in affected patients. This review provides a foundation for future research and a call to action for the global health community to prioritize recognition and management of this serious dengue complication.

## Figures and Tables

**Table 1:** Characteristics of patients with HLH due to dengue.

Characteristic	All Patients (*n* = 133) *	Lived (*n* = 88) *	Died (*n* = 21) *	*p*-Value
Age, years, median (IQR)	18 (8–33.8)	25 (14–36.5)	19 (6.5–40.5)	0.4653
Male gender, *n* (%)	75/132 (56.8)	54 (61.4)	9 (42.9)	0.1447
Predisposing factors				
Immunosuppression, *n* (%)	3/105 (2.9)	1/64 (1.6)	2/17 (11.8)	0.11
Previous HLH, *n* (%)	1/105 (0.9)	1/64 (1.6)	0/17 (0)	1
Clinical characteristics				
Fever, *n* (%)	125/129 (96.9)	80/84 (95.2)	21 (100)	0.5814
Poor feeding/anorexia, *n* (%)	17/93 (18.3)	12/73 (16.4)	5/18 (27.8)	0.3141
Jaundice, *n* (%)	11/93 (11.8)	4/73 (5.5)	7/18 (38.9)	0.0008
Organomegaly, *n* (%)	62/95 (65.3)	50/75 (66.7)	11/18 (61.1)	0.7832
Lymphadenopathy, *n* (%)	6/95 (6.3)	5/75 (6.7)	1/18 (5.6)	1
Lethargy/drowsiness, *n* (%)	18/93 (19.4)	11/73 (15.1)	7/18 (38.9)	0.0425
Respiratory symptoms, *n* (%)	31/93 (33.3)	22/73 (30.1)	8/18 (44.4)	0.2722
Fatigue/weakness, *n* (%)	29/93 (31.2)	24/73 (32.9)	5/18 (27.8)	1
Bleeding, *n* (%)	23/93 (24.7)	16/73 (21.9)	5/18 (27.8)	0.7828
Skin rash, *n* (%)	28/93 (30.1)	23/73 (31.5)	4/18 (22.2)	0.5697
Neurological symptoms, *n* (%)	11/93 (11.8)	7/73 (9.6)	4/18 (22.2)	0.2180
Gastrointestinal symptoms, *n* (%)	35/93 (37.6)	30/73 (41.1)	5/18 (27.8)	0.4188
Abdominal pain, *n* (%)	31/93 (33.3)	27/73 (37)	4/18 (22.2)	0.2794
Laboratory characteristics				
White blood cells K/mL, median (IQR)	2900 (1800–4500)	2500 (1700–4350)	3600 (2950–9450)	0.0459
Hemoglobin g/dL, median (IQR)	8.9 (7.4–10.9)	9 (7.7–11.4)	8.1 (7–9.9)	0.1996
Platelets K/mL, median (IQR)	30,000 (19,000–44,500)	28,000 (18,000–40,000)	44,500 (21,950–68,675)	0.0995
AST U/mL, median (IQR)	966 (188.5–2144)	919 (198–1886)	2390 (363–11,921)	0.1586
ALT U/mL, median (IQR)	408.5 (197.8–1220)	382 (186.5–777)	3074 (1283–7834)	0.0002
LDH U/mL, median (IQR)	1118 (500.8–2073)	1082 (470–1822)	5166 (390.3–7585)	0.3891
Triglycerides md/dL, median (IQR)	295 (234–398.3)	295 (217.5–385.8)	283.4 (217.8–590)	0.9116
C-reactive protein mg/dL, median (IQR)	33 (5–86.4)	24 (4.1–54)	52.2 (12.3–92)	0.6018
Fibrinogen mg/dL, median (IQR)	155.9 (109.3–205.8)	170.8 (120.8–203.3)	100 (67.6–135)	0.0045
Ferritin ng/dL, median (IQR)	28,060 (9270–75,031)	34,951 (9600–93,026)	40,000 (18,250–93,273)	NA
Dengue type				
DENV-1, *n* (%)	27/47 (57.4)	16/22 (72.7)	2/8 (25)	0.0342
DENV-2, *n* (%)	3/47 (6.4)	2/22 (9.1)	1/8 (12.5)	1
DENV-3, *n* (%)	8/47 (17)	6/22 (18.2)	4/8 (50)	0.1580
DENV-4, *n* (%)	9/47 (19.1)	0/22 (0)	1/8 (12.5)	0.2667
Polymicrobial, *n* (%)	15/112 (13.4)	9/71 (12.7)	6/18 (33.3)	0.0708
Complications				
Sepsis, *n* (%)	56/104 (53.8)	37/81 (45.7)	18 (85.7)	0.0011
Organ dysfunction, *n* (%)	54/105 (51.4)	36/82 (43.9)	17 (81)	0.003
Shock, *n* (%)	30/98 (30.6)	16/77 (20.8)	13/19 (68.4)	0.0001
Need for ICU, *n* (%)	21/90 (23.3)	8/72 (11.1)	13/16 (81.3)	<0.0001
Treatment				
Empirical antibiotic therapy, *n* (%)	31/108 (28.7)	21/87 (24.1)	9/19 (47.4)	0.0522
IVIG, *n* (%)	24/110 (21.8)	22/88 (25)	2 (9.5)	0.1523
Cyclosporin A, *n* (%)	3/106 (2.8)	3/84 (3.6)	0 (0)	1
Etoposide, *n* (%)	7/106 (6.6)	5/84 (6)	2 (9.5)	0.625
Steroids, *n* (%)	68/106 (64.2)	56/84 (66.7)	11 (52.4)	0.4361
Fresh frozen plasma, *n* (%)	19/104 (18.3)	13/82 (15.9)	6 (28.6)	0.2105
Plasma exchange, *n* (%)	5/104 (4.8)	2/82 (2.4)	3 (14.3)	0.0563
Outcomes				
Deaths overall, *n* (%)	21/109 (19.3)	NA	NA	NA

DENV1/2/3/4: dengue virus-1/2/3/4; HLH: hemophagocytic lymphohistiocytosis; IQR: interquartile range; IVIG: intravenous immunoglobulin; NA: not applicable *: data are among the number of patients mentioned on top unless otherwise described.

## Data Availability

Not applicable.

## References

[1] Sen E.S., Clarke S.L.N., Ramanan A.V. (2016). Macrophage Activation Syndrome. Indian J. Pediatr..

[2] Otrock Z.K., Daver N., Kantarjian H.M., Eby C.S. (2017). Diagnostic Challenges of Hemophagocytic Lymphohistiocytosis. Clin. Lymphoma Myeloma Leuk..

[3] Machowicz R., Janka G., Wiktor-Jedrzejczak W. (2017). Similar but Not the Same: Differential Diagnosis of HLH and Sepsis. Crit. Rev. Oncol. Hematol..

[4] Al-Samkari H., Berliner N. (2018). Hemophagocytic Lymphohistiocytosis. Annu. Rev. Pathol..

[5] Ponnatt T.S., Lilley C.M., Mirza K.M. (2022). Hemophagocytic Lymphohistiocytosis. Arch. Pathol. Lab. Med..

[6] Ong L.T., Balasubramaniam R. (2024). Prevalence and Mortality of Haemophagocytic Lymphohistiocytosis in Dengue Fever: A Systematic Review and Meta-Analysis. Trans. R. Soc. Trop. Med. Hyg..

[7] Roy S.K., Bhattacharjee S. (2021). Dengue Virus: Epidemiology, Biology, and Disease Aetiology. Can. J. Microbiol..

[8] Kothari D., Patel N., Bishoyi A.K. (2025). Dengue: Epidemiology, Diagnosis Methods, Treatment Options, and Prevention Strategies. Arch. Virol..

[9] Henter J.-I., Horne A., Aricó M., Egeler R.M., Filipovich A.H., Imashuku S., Ladisch S., McClain K., Webb D., Winiarski J. (2007). HLH-2004: Diagnostic and Therapeutic Guidelines for Hemophagocytic Lymphohistiocytosis. Pediatr. Blood Cancer.

[10] Henter J.-I. (2025). Hemophagocytic Lymphohistiocytosis. N. Engl. J. Med..

[11] Wong K.F., Chan J.K.C., Chan J.C.W., Lim W.W.L., Wong W.K. (1991). Letter to the Editor: Dengue Virus Infection-associated Hemophagocytic Syndrome. Am. J. Hematol..

[12] Lu P., Hsiao H., Tsai J., Chen T., Chen T., Lin S., Feng M. (2005). Dengue Virus-Associated Hemophagocytic Syndrome and Dyserythropoiesis: A Case Report. Kaohsiung J. Med. Sci..

[13] Jain D., Singh T. (2008). Dengue Virus Related Hemophagocytosis: A Rare Case Report. Hematology.

[14] Srichaikul T., Punyagupta S., Kanchanapoom T., Chanokovat C., Likittanasombat K., Leelasiri A. (2008). Hemophagocytic Syndrome in Dengue Hemorrhagic Fever with Severe Multiorgan Complications. J. Med. Assoc. Thai..

[15] Vijayalakshmi A.M., Ganesh V.R.R. (2009). Hemophagocytic Syndrome Associated with Dengue Hemorrhagic Fever. Indian Pediatr..

[16] Joshi R., Phatarpekar A., Currimbhoy Z., Desai M. (2011). Haemophagocytic Lymphohistiocytosis: A Case Series from Mumbai. Ann. Trop. Paediatr..

[17] Ray S., Kundu S., Saha M., Chakrabarti P. (2011). Hemophagocytic Syndrome in Classic Dengue Fever. J. Glob. Infect. Dis..

[18] Kapdi M., Shah I. (2012). Dengue and Haemophagocytic Lymphohistiocytosis. Scand. J. Infect. Dis..

[19] Tangnararatchakit K., Tirapanich W., Tapaneya-Olarn W., Sumethkul V., Sirachainan N., Watcharananan S., Leenanupunth C., Yoksan S., Chuansumrit A. (2012). Severe Nonfebrile Dengue Infection in an Adolescent After Postoperative Kidney Transplantation: A Case Report. Transpl. Proc..

[20] Tan L.H., Lum L.C.S., Omar S.F.S., Kan F.K. (2012). Hemophagocytosis in Dengue: Comprehensive Report of Six Cases. J. Clin. Virol..

[21] Nair V., Das S., Sharma A., Sharma S., Sharma P., Ray S., Bhattacharya S. (2013). A Clinicopathological Analysis of 26 Patients with Infection-Associated Haemophagocytic Lymphohistiocytosis and the Importance of Bone Marrow Phagocytosis for the Early Initiation of Immunomodulatory Treatment. Postgrad. Med. J..

[22] Morel Z., Ramírez A. (2014). Respuesta autoinmune en niños con dengue. Reporte de casos. Reumatol. Clínica.

[23] Sharp T.M., Gaul L., Muehlenbachs A., Hunsperger E., Bhatnagar J., Lueptow R., Santiago G.A., Muñoz-Jordan J.L., Blau D.M., Ettestad P. (2014). Fatal Hemophagocytic Lymphohistiocytosis Associated with Locally Acquired Dengue Virus Infection—New Mexico and Texas, 2012. MMWR. Morb. Mortal. Wkly. Rep..

[24] Mitra S., Bhattacharyya R. (2014). Hemophagocytic Syndrome in Severe Dengue Fever: A Rare Presentation. Indian J. Hematol. Blood Transfus..

[25] De Koninck A.-S., Dierick J., Steyaert S., Taelman P. (2014). Hemophagocytic Lymphohistiocytosis and Dengue Infection: Rare Case Report. Acta Clin. Belg..

[26] Ribeiro E., Kassab S., Pistone T., Receveur M.-C., Fialon P., Malvy D. (2014). Primary Dengue Fever Associated with Hemophagocytic Syndrome: A Report of Three Imported Cases, Bordeaux, France. Intern. Med..

[27] Kodan P., Chakrapani M., Shetty M., Pavan R., Bhat P. (2015). Hemophagocytic Lymphohistiocytosis Secondary to Infections: A Tropical Experience!. J. Postgrad. Med..

[28] Hein N., Bergara G.H., Moura N.B.V., Cardoso D.M., Hirose M., Ferronato A.E., Pastorino A.C., Lo D.S., Gilio A.E. (2015). Dengue Fever as a Cause of Hemophagocytic Lymphohistiocytosis. ACR.

[29] Arshad U., Ahmad S.Q., Khan F. (2015). Hemophagocytic Lymphohistiocytosis in a Patient with Dengue Infection. Hematol. Oncol. Stem Cell Ther..

[30] Khurram M., Faheem M., Umar M., Yasin A., Qayyum W., Ashraf A., Zahid Khan J., Hasnain Yasir A., Ansari Y., Asad M. (2015). Hemophagocytic Lymphohistiocytosis Complicating Dengue and *Plasmodium vivax* Coinfection. Case Rep. Med..

[31] Phuakpet K., Sanpakit K., Vathana N., Takpradit C., Chokephaibulkit K., Viprakasit V. (2015). Hemophagocytic Lymphohistiocytosis Following Dengue Hemorrhagic Fever in H b H/H b Constant Spring Patient. Pediatr. Int..

[32] Wan Jamaludin W.F., Periyasamy P., Wan Mat W.R., Abdul Wahid S.F. (2015). Dengue Infection Associated Hemophagocytic Syndrome: Therapeutic Interventions and Outcome. J. Clin. Virol..

[33] Kobayashi K., Hikone M., Sakamoto N., Iwabuchi S., Kashiura M., Takasaki T., Fujita H., Ohnishi K. (2015). Dengue-Associated Hemophagocytic Syndrome in a Japanese Traveler: A Case Report. J. Travel. Med..

[34] Ab-Rahman H.A., Wong P.-F., Rahim H., Abd-Jamil J., Tan K.-K., Sulaiman S., Lum C.-S., Syed-Omar S.-F., AbuBakar S. (2015). Dengue Death with Evidence of Hemophagocytic Syndrome and Dengue Virus Infection in the Bone Marrow. SpringerPlus.

[35] Ellis E.M., Sharp T.M., Pérez-Padilla J., González L., Poole-Smith B.K., Lebo E., Baker C., Delorey M.J., Torres-Velasquez B., Ochoa E. (2016). Incidence and Risk Factors for Developing Dengue-Associated Hemophagocytic Lymphohistiocytosis in Puerto Rico, 2008–2013. PLoS Negl. Trop. Dis..

[36] Lakhotia M., Pahadiya H.R., Gandhi R., Prajapati G.R., Choudhary A. (2016). Stuck with Pancytopenia in Dengue Fever: Evoke for Hemophagocytic Syndrome. Indian J. Crit. Care Med..

[37] Koshy M., Mishra A.K., Agrawal B., Kurup A.R., Hansdak S.G. (2016). Dengue Fever Complicated by Hemophagocytosis. Oxf. Med. Case Rep..

[38] Nandi M., Roy S., Das M., Datta C. (2016). Neonatal-Onset Hemophagocytic Lymphohistiocytosis Associated with Primary Dengue Infection. Med. J. DY Patil. Univ..

[39] Anam A.M., Rabbani R., Shumy F. (2017). Expanded Dengue Syndrome: Three Concomitant Uncommon Presentations in the Same Patient. Trop. Doct.

[40] Chung S.M., Song J.Y., Kim W., Choi M.J., Jeon J.H., Kang S., Jung E., Noh J.Y., Cheong H.J., Kim W.J. (2017). Dengue-Associated Hemophagocyticlymphohistiocytosis in an Adult: A Case Report and Literature Review. Medicine.

[41] Ray U., Dutta S., Mondal S., Bandyopadhyay S. (2017). Severe Dengue Due to Secondary Hemophagocytic Lymphohistiocytosis: A Case Study. IDCases.

[42] Krithika M., Amboiram P., Latha S.M., Ninan B., Suman F.R., Scott J. (2017). Neonate with Haemophagocytic Lymphohistiocytosis Secondary to Dengue Infection: A Case Report. Trop. Dr..

[43] Jasmine Y.S.Y., Lee S.L., Kan F.K. (2017). Infection Associated Haemophagocytic Syndrome in Severe Dengue Infection—A Case Series in a District Hospital. Med. J. Malays..

[44] Kam K., Soh S.Y., Bhattacharyya R. (2018). Dengue-Associated Hemophagocytic Lymphohistiocytosis: A Rare Complication of a Common Infection in Singapore. J. Pediatr. Hematol. Oncol..

[45] Ray U., Dutta S., Bandyopadhyay S., Mondal S. (2019). Infections and HLH—Experience from a Tertiary Care Centre. J. Assoc. Physicians India.

[46] Mushtaque R.S., Ahmad S.M., Mushtaque R., Baloch S. (2020). A Curious Case of Dengue Fever: A Case Report of Unorthodox Manifestations. Case Rep. Med..

[47] Thadchanamoorthy V., Dayasiri K. (2020). Dengue Fever Associated Haemophagocytic Lymphohistiocytosis: A Report of Two Children. Cureus.

[48] Narayanasami E., Umakanth M., Suganthan N. (2020). Dengue Hemorrhagic Fever Complicated with Hemophagocytic Lymphohistiocytosis in an Adult with Diabetic Ketoacidosis. Cureus.

[49] Ishak S.H., Yaacob L.H., Ishak A. (2020). Severe Dengue with Hemophagocytosis Syndrome. Malays. Fam. Physician.

[50] Agrawal G., Wazir S., Sachdeva A., Kumar S. (2020). Primary Dengue Infection Triggered Haemophagocytic Lymphohistiocytosis in a Neonate. BMJ Case Rep..

[51] Chang C.Y., Rajappan M., Zaid M., Ong E.L.C. (2020). Dengue Fever Complicated by Hemophagocytic Lymphohistiocytosis: Report of 2 Cases and Bone Marrow Findings. Clin. Case Rep..

[52] Kanitkar T., Richardson C., Scobie A., Ireson A., Singh A., Jacobs M., Buckley J., Spiro M. (2020). Fatal Primary Dengue-Induced Haemophagocytic Lymphohistiocytosis (HLH) in a Returning Traveller from India Treated with Anakinra for the First Time. Clin. Infect. Pract..

[53] Takkinsatian P., Sowithayasakul P., Prommalikit O. (2020). Dengue Associated Haemophagocytic Lymphohystiocytosis: An Often-Missed Complication of a Common Infection. Med. J. Malays..

[54] Islam Q.T., Sagor H.B., Tuli T.C. (2020). Haemophagocytic Lymphohistiocytosis Associated with Dengue Fever—A Case Series. J. Med..

[55] Munshi A., Alsuraihi A., Balubaid M., Althobaiti M., Althaqafi A. (2021). Dengue-Induced Hemophagocytic Lymphohistiocytosis: A Case Report and Literature Review. Cureus.

[56] Chang C.Y. (2021). A Fatal Case of Dengue-Associated Hemophagocytic Lymphohistiocytosis and Retroperitoneal Hematoma in a Patient With Autoimmune Hemolytic Anemia. Cureus.

[57] Krishnappa A., Munusamy J., Ray S., Rameshbabu M., Bhatia P., Roy P.S., Sundaram V., Kumar P. (2021). Neonatal Dengue with HLH: Perks of Early Diagnosis and Management. J. Pediatr. Hematol. Oncol..

[58] Devi K., Ali N. (2021). Case Report: Primary Hemophagocytic Syndrome Triggered by Dengue Infection. IDCases.

[59] Kumar H.C.K., Kumar K.J., Balaji S. (2021). Hemophagocytic Lymphohistiocytosis Associated with Coinfection of Scrub Typhus and Dengue Fever in a Child: A Case Report. Mediterr. J. Infect. Microb. Antimicrob..

[60] Cheo S., Abdul Rashid W., Ho C., Ahmad Akhbar R.Z., Low Q., Rajahram G.S. (2021). Haemophagocytic Lymphohistiocytosis Secondary to Dengue Fever: A Case Report. Hong Kong Med. J..

[61] Jose P.-M.M., Paola Z.-S., Eduardo D.-G., Arturo S.-M.M.O., Fernando B.-G. (2021). A Case of Coinfection of a Pediatric Patient with Acute SARS-CoV-2 with MIS-C and Severe DENV-2 in Mexico: A Case Report. BMC Infect. Dis..

[62] Kamineni M., Pai T., D’Sa S., Bhat K. (2020). Acute Dengue Fever in a Neonate Secondary to Perinatal Transmission. Indian J. Nephrol..

[63] Saw Y.T., Lee H.G. (2021). Concurrent COVID-19 and Dengue with Hyperferritinaemia: A Case Report. Med. J. Malays..

[64] Ren D., Ong S.W.X., Batac J.A.L., Fan B.E., Vasoo S. (2021). Haemophagocytic Lymphohistiocytosis in Dengue Fever. Lancet Infect. Dis..

[65] Acharya S., Shukla S., Sontakke T., Vs I., Bagga C., Dronamraju S., Giri A. (2022). A Case Report of Hemophagocytic Lymphohistiocytosis (HLH)—An Unusual Complication of Dengue Infection. Cureus.

[66] Srivatsav S., Mahalingam S., Ramineni P., Manya S. (2022). Dengue and Plasmodium Falciparum Coinfection with Secondary Hemophagocytic Lymphohistiocytosis in a 3-Year-Old Boy: A Clinical Conundrum. J. Pediatr. Hematol. Oncol..

[67] Jha V.K., Khurana H., Balakrishnan A. (2022). Prolonged Fever and Pancytopenia in a Case of Severe Dengue May Be Secondary Hemophagocytic Lymphohistiocytosis. Med. J. Armed Forces India.

[68] Porel R., Kumar V., Agarwal K., Biswas R., Ojha V.S. (2023). Secondary Hemophagocytic Lymphohistiocytosis: A Series of Three Cases. Cureus.

[69] Ray S., Kumar M., Mahajan N., Khatri A. (2023). Paediatric Hemophagocytic Lymphohistiocytosis: A Case Series with a Diverse Spectrum from a Resource-Limited Setting. Cureus.

[70] Krishnan G., Gosavi S., Gujral M., Basheer N., Kumar B., Jain P. (2023). Hemophagocytic Lymphohistiocytosis: A Scourge for the Physician and Bane to the Bone Marrow. Ann. Afr. Med..

[71] Arora A., Verma S., Khot N., Chalipat S., Agarkhedkar S., Kiruthiga K.G. (2023). A Case Report on CNS Hemophagocytic Lymphohistiocytosis in an Infant with Dengue Hemorrhagic Fever. Cureus.

[72] Mizutani N., Kenzaka T., Nishisaki H. (2023). Dengue Fever Complicated with Hemophagocytic Lymphohistiocytosis: A Case Report of Resolution with Steroid-Sparing Supportive Care. TropicalMed.

[73] Pradeep C., Karunathilake P., Abeyagunawardena S., Ralapanawa U., Jayalath T. (2023). Hemophagocytic Lymphohistiocytosis as a Rare Complication of Dengue Haemorrhagic Fever: A Case Report. J. Med. Case Rep..

[74] Shankar M., Gurusiddiah S.C., Nayaka M., Aralapuram K. (2023). An Uncommon Complication of a Common Tropical Infection in a Kidney Transplant Recipient—A Case Report. IJN.

[75] Kazi A.N., Ahmed M., Wasim M.A., Abbasi L.I., Herekar F.F., Patel M.J. (2024). A Vector Borne, Airborne and Food Borne Infection with Secondary Hemophagocytic Lymphohistocytosis: Case of Triple Infections in an Immuno-Competent Patient. Indian J. Med. Microbiol..

[76] Dinkar A., Singh J., Kumar N., Kumar K., Singh S.K. (2024). Dengue-Related Hemophagocytic Lymphohistiocytosis in an Adult: A Case Report and Brief Update. Avicenna J. Med..

[77] Raza M., Ali S. (2024). Hemophagocytic Lymphohistiocytosis (HLH): A Rare Complication of Dengue Hemorrhagic Fever. Cureus.

[78] Ramamoorthy L., Sivakumar N., Murugesan L., Kumar A. (2024). Hemophagocytic Lymphohistiocytosis Secondary to Dengue Fever in a Pediatric Patient: A Case Report. Cureus.

[79] Mayurathan P. (2024). Dengue Hemorrhagic Fever Causing Postpartum Hemorrhage and Hemophagocytic Lymphohistiocytosis in a Young Woman: A Case Report. Cureus.

[80] Mateen S., Mishra A., Singh S., Jabeen F. (2025). Severe Dengue, Aneurysmal Sub-Arachnoid Hemorrhage, and Hemophagocytic Lymphohistiocytosis: A Rare Case Combination. Einstein.

[81] Sharma R., Ray S., Chattopadhyay A., Khatri A. (2024). Dengue-Associated Hemophagocytic Lymphohistiocytosis: The Many Faces of Expanded Dengue Syndrome. Clin. Pediatr..

[82] La Marle S., Richard-Colmant G., Fauvernier M., Ghesquières H., Hot A., Sève P., Jamilloux Y. (2023). Mortality and Associated Causes in Hemophagocytic Lymphohistiocytosis: A Multiple-Cause-of-Death Analysis in France. JCM.

[83] Annan E., Treviño J., Zhao B., Rodriguez-Morales A.J., Haque U. (2023). Direct and Indirect Effects of Age on Dengue Severity: The Mediating Role of Secondary Infection. PLoS Negl. Trop. Dis..

[84] Sohail A., Zhong S., Nguyen P.-Y., McGuinness S.L., Leder K. (2024). Dengue Fever in Immunocompromised Patients: A Systematic Review and Meta-Analysis. Int. J. Infect. Dis..

[85] See K.C. (2024). Dengue-Associated Hemophagocytic Lymphohistiocytosis: A Narrative Review of Its Identification and Treatment. Pathogens.

[86] Tuiskunen Bäck A., Lundkvist Å. (2013). Dengue Viruses—An Overview. Infect. Ecol. Epidemiol..

[87] Murray N.E.A., Quam M.B., Wilder-Smith A. (2013). Epidemiology of Dengue: Past, Present and Future Prospects. Clin. Epidemiol..

[88] Cattaneo P., Salvador E., Manica M., Barzon L., Castilletti C., Di Gennaro F., Huits R., Merler S., Poletti P., Riccardo F. (2025). Transmission of Autochthonous Aedes-Borne Arboviruses and Related Public Health Challenges in Europe 2007–2023: A Systematic Review and Secondary Analysis. Lancet Reg. Health—Eur..

[89] Buchs A., Conde A., Frank A., Gottet C., Hedrich N., Lovey T., Shindleman H., Schlagenhauf P. (2022). The Threat of Dengue in Europe. New Microbes New Infect..

[90] Masood M., Siddique A., Krishnamoorthi R., Kozarek R.A. (2024). Liver Dysfunction in Adult Hemophagocytic Lymphohistiocytosis: A Narrative Review. Adv. Ther..

[91] Kohli S., Chadha R., Rastogi N., Yadav S.P. (2021). High Serum Ferritin Alone as a Predictor of Mortality and Hemophagocytic Lymphohistiocytosis. eJHaem.

[92] La Rosée P., La Rosée F. (2024). HLH: Diagnostics Revisited and Improved. Blood.

[93] Raafat N., Blacksell S.D., Maude R.J. (2019). A Review of Dengue Diagnostics and Implications for Surveillance and Control. Trans. R. Soc. Trop. Med. Hyg..

[94] Jordan M.B., Allen C.E., Weitzman S., Filipovich A.H., McClain K.L. (2011). How I Treat Hemophagocytic Lymphohistiocytosis. Blood.

[95] La Rosée P., Horne A., Hines M., Von Bahr Greenwood T., Machowicz R., Berliner N., Birndt S., Gil-Herrera J., Girschikofsky M., Jordan M.B. (2019). Recommendations for the Management of Hemophagocytic Lymphohistiocytosis in Adults. Blood.

[96] Adrizain R., Setiabudi D., Chairulfatah A. (2019). The Inappropriate Use of Antibiotics in Hospitalized Dengue Virus-Infected Children with Presumed Concurrent Bacterial Infection in Teaching and Private Hospitals in Bandung, Indonesia. PLoS Negl. Trop. Dis..

[97] Giang H.T.N., Banno K., Minh L.H.N., Trinh L.T., Loc L.T., Eltobgy A., Tai L.L.T., Khan A., Tuan N.H., Reda Y. (2018). Dengue Hemophagocytic Syndrome: A Systematic Review and Meta-Analysis on Epidemiology, Clinical Signs, Outcomes, and Risk Factors. Rev. Med. Virol..

[98] Kan F.K., Tan C.C., Von Bahr Greenwood T., Khalid K.E., Supramaniam P., Hed Myrberg I., Tan L.H., Henter J.-I. (2020). Dengue Infection Complicated by Hemophagocytic Lymphohistiocytosis: Experiences from 180 Patients with Severe Dengue. Clin. Infect. Dis..

